# A case of ovarian mesothelioma with VHL mutation diagnosed by immunohistochemistry and literature review

**DOI:** 10.3389/fonc.2024.1471978

**Published:** 2024-11-13

**Authors:** Ye Yao, Hao Yan, Jing Xiong, Yaqi Duan

**Affiliations:** ^1^ Department of Nephrology, Union Hospital, Tongji Medical College, Huazhong University of Science and Technology, Wuhan, China; ^2^ Department of Gynecologic Oncology, Hubei Cancer Hospital, Wuhan, Hubei, China; ^3^ Department of Pathology, Tongji Hospital, Tongji Medical College, Huazhong University of Science and Technology, Wuhan, China

**Keywords:** ovarian mesothelioma, ovarian clear cell tumor, differential diagnosis, VHL gene, immunohistochemistry

## Abstract

The malignant mesothelioma mainly develops in the pleura and peritoneum, while primary ovarian mesothelioma is very rare. Here, we report the first case of primary ovarian mesothelioma (clear cell variant) with VHL mutations in the world based on the results of histomorphology, immunohistochemistry, and genetic testing. This is an extremely rare type of tumor that has not been reported so far. Through the literature search, we reviewed primary ovarian mesothelioma, focusing on its differential diagnosis and molecular genetics. The purpose of this paper is to deepen the flexible selection and application of immunohistochemical markers in mesothelioma, so as to reduce missed diagnosis and misdiagnosis.

## 1 Background

Malignant mesothelioma(MM) originates from the mesothelium, which is a single flat epithelium distributed on the surface of the pleura, peritoneum, and pericardium, mainly occurring in the pleura and peritoneum, while primary ovarian malignant mesothelioma (OMM) is very rare. The first report of malignant mesothelioma was made by Miller and Wynn in 1908 (1), and it was not until 1983 that Addis and Fox proposed the concept of “primary ovary” (2). Compared with epithelial ovarian tumors in gynecology, OMM is difficult to identify. Due to its diverse morphology and great heterogeneity, it is easy to be confused with other tumors under hematoxylin and eosin staining. According to histological characteristics, mesothelioma can be divided into three basic types: epithelioid, sarcomatoid, and biphasic (mixed type). Epithelioid MM is the most common type and includes polygonal, oval, cubic, clear cell-like, and signet-ring-like subtypes (3, 4). The clear cell variant of mesothelioma is very rare. The earliest one is a case of clear cell variant of pleural mesothelioma reported by Nelson G. Ordóñez in 1996 (5). Subsequent reports were limited to the peritoneal, peritonea, uterus, and testes scabbard film (6–9). There has been no report on OMM with clear cell variants so far. After a complex and tortuous diagnostic process, taking into full consideration the results of histology, immunohistochemistry, and gene sequencing, and conducting multi-team consultations, we hereby report the first case of primary ovarian mesothelioma (clear cell variant) with Von Hippel–Lindau (VHL) gene mutation in the world.

## 2 Case report

### 2.1 Initial diagnosis and treatment

**Figure 1:**
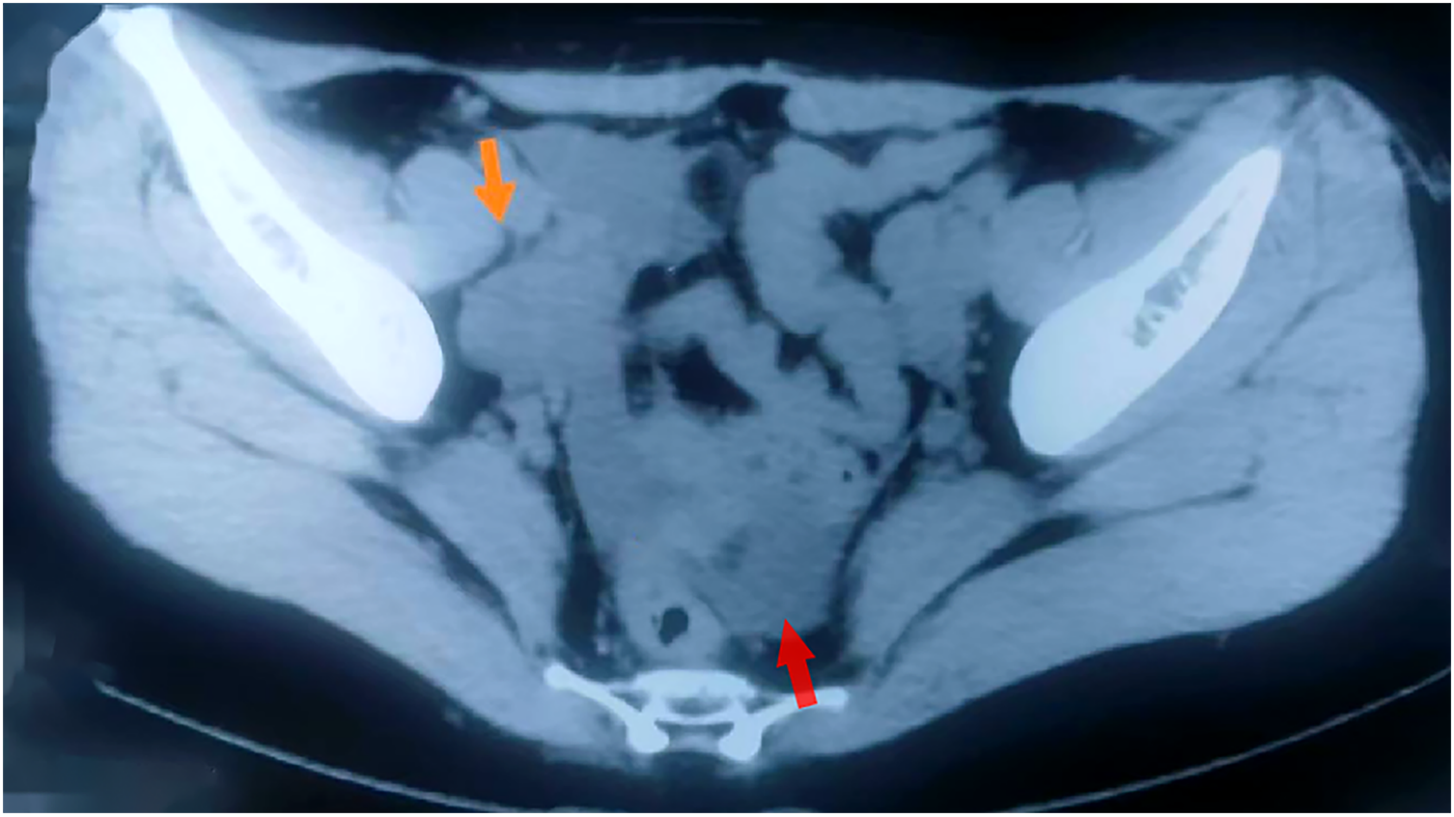
Imaging of the tumor. CT showed an isodense mass in the right ovary (orange arrow), with a small amount of fluid in the Douglas cavity (red arrow).

Early April 2022, a 40-year-old female patient was diagnosed with right ovarian isodensity occupying lesion by computed tomography (CT) in a local hospital (**Figure 1**), and serum carbohydrate antigen 125 (CA125) was slightly elevated (62 U/ml). The patient had no special menstrual history, no family history of tumors, and no history of asbestos exposure. After weighing the benefits and drawbacks, the patient agreed to have surgery at the local hospital. During the surgery, the omentum was found to be widely adherent to the pelvis and abdominal wall. The uterus was normal in size and attached to the bladder, abdominal wall, and intestinal canal. The pelvic cavity contained approximately 300 ml of pale-yellow liquid. The right ovary measured 5 cm × 4 cm × 4 cm. It had a smooth exterior and some hard parts. No obvious abnormality in the appearance of the bilateral fallopian tubes or the left ovary was observed. The quick freezing of intraoperative specimens revealed an ovarian tumor on the right side. A borderline ovarian tumor was highly probable, and the presence of a low-grade malignant tumor could not be excluded. Consequently, a comprehensive staging operation for the ovarian tumors was performed: total hysterectomy + bilateral adnophorectomy + pelvic lymph node dissection + para-aortic lymph node dissection + bilateral ovarian artery and vein high ligation + omentum resection.

### 2.2 Histological

**Figure 2:**
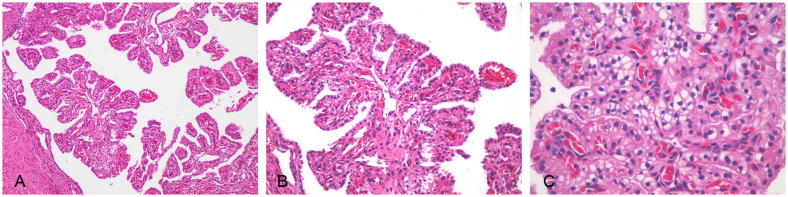
Ovarian malignant mesothelioma was seen in hematoxylin–eosin-stained sections. The tumor cells had small atypia; no obvious mitosis, focal well-differentiated papillary mesothelial tumor conformation, complex papillary structure, and interstitial infiltration of the axis of the papilla were observed in some areas. **(A)** Papillary structure (×100). **(B)** Tumor cells were cubic and oval (×200). **(C)** Tumor cells were uniform in size, with clear cytoplasm, small atypia, and no obvious mitoses (×400).

The postoperative pathological examination showed that the right ovarian tumor was 2 cm in diameter and located in the ovary, with a diameter of 0.5 cm nodule in the greater omentum. The boundary of the two lesions was clear, the tumor cells were small, and there was no obvious nuclear division. No tumor tissue was found in the uterus, left adnexa, right fallopian tube, vaginal fornies, and pelvic lymph nodes (**Figure 2**).

### 2.3 Similarities and differences of three immunohistochemical tests

**Figure 3:**
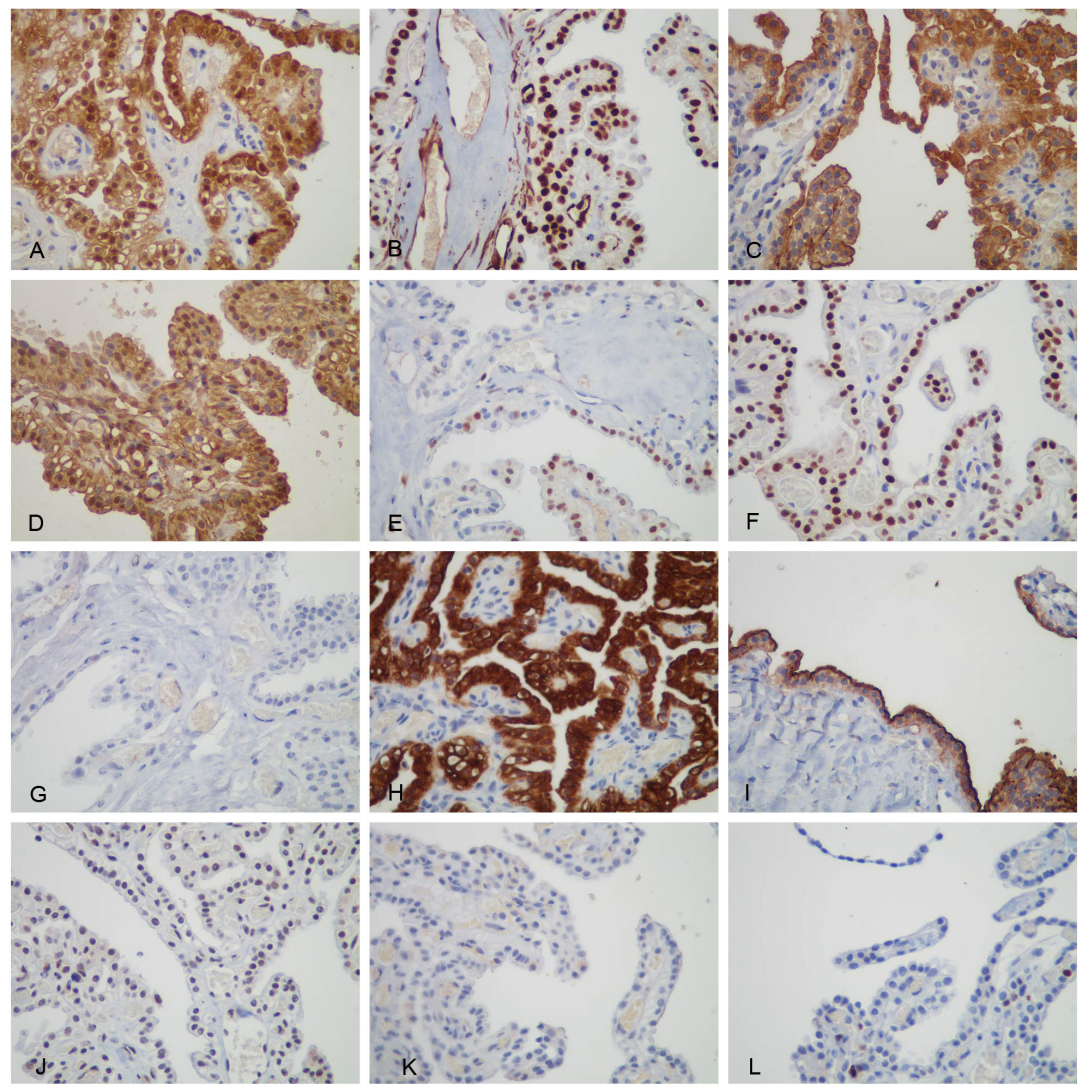
Immunohistochemical staining of the tumor cells. **(A)** Both cytoplasmic and nuclear were positive for Calretinin, **(B)** only nuclear was positive for WT-1, **(C)** membranous was positive for D2-40, **(D)** both cytoplasmic and nuclear were positive for Vimentin, **(E)** nuclear was positive for GATA-3 positive, **(F)** nuclear was positive for PAX-8, **(G)** TTF-1, **(H)** membranous was positive for CK5/6, **(I)** membranous was positive for mesothelin (HBME -1), **(J)** nuclear was weakly positive for BAP1, **(K)** Napsin A, and **(L)** Ki-67 positive rate of approximately 5%.

The immunohistochemical results of postoperative specimens from local hospitals were as follows: Calretinin (partly+), Wilms tumor gene-1 (WT-1) (−), D2-40 (+), Vimentin (+), GATA-3 (partly+), Paired box gene 8 (PAX-8) (+), Estrogen receptor (ER) (−), Progesterone receptor (PR) (−), Arcinoembryonic antigen (CEA) (−), and Thyroid transcription factor-1 (TTF-1) (−) (**Figure 3**). Considering the clear cell appearance of the ovarian tumor with positive PAX-8 nuclei and negative WT-1, a diagnosis of ovarian clear cell carcinoma was made in the local hospital.

Nonetheless, some MM markers showed positive results (Calretinin, D2-40, and GATA-3). The diagnosis was still uncertain; therefore, pathological consultation was performed in another hospital, the Affiliated Hospital of Tongji Medical College. On April 17, another hospital reported that the re-staining of WT-1 was positive (the local hospital was negative), and four new markers were added. They included CK5/6(+), Hector Battifora Mesothelial-1 (HBME-1) (+), BRCA-associated protein 1 (BAP1) (weakly +), and NapsinA(−) (**Figure 3**), indicating a high possibility of mesothelioma.

The diagnosis differed between the two hospitals, and the patient was further seen at a Shanghai hospital in March of the following year. Three new markers were stained by immunohistochemistry: MOC31 (+), Ber-EP4 (+), and hepatocyte nuclear factor-1β (HNF-1β) (−). In addition, further gene testing using a targeted next generation sequencing showed that the first exon of the VHL gene was mutated, p. N78S (c.233A>G) (mutant abundance, 29.36%), p.S65L (c.194C>T) (27.38%). The diagnosis was changed to clear cell papillary cystadenoma of the ovary, related to VHL syndrome.

In view of the twists and turns in the pathological diagnosis (**Table 1**), the patient returned to the Affiliated Hospital of Tongji Medical College. After carefully reading the pathological slides and searching the literature, the multidisciplinary treatment from the departments of pathology, gynecology, oncology, and other related departments concluded that the specificity of PAX-8, MOC-31, and Ber-EP4 in mesothelioma was not 100%, while the patient was positive for multiple MM markers. The immunohistochemical results of the patient were consistent with the diagnosis of mesothelioma, and the final diagnosis was ovarian mesothelioma (clear cell type) with VHL gene mutation.

**Table 1:** IHC marker testing institution and time.

Hospital	Time	Positive markers in support of MM	Negative markers in support of MM	Positive markers of MM were excluded	Negative markers of MM were excluded	Diagnosis
The local hospital	2022.4.10	D2-40(+), Calretinin(partly+), GATA-3(partly+)	ER(−), PR(−), TTF-1(−), CEA(−)	PAX-8(+)	WT-1 (−)*	Ovarian clear cell carcinoma
The Affiliated Hospital of Tongji Medical College^#^	2022.4.17	WT-1(+)*, CK5/6(+), mesothelin(+), Calretinin(+), GATA-3(+)	NapsinA(−)	BAP1(weakly+)		Ovarian malignant mesothelioma
A Shanghai hospital^#^	2023.3		HNF-1β(−)	MOC-31(+), Ber-EP4(+)		Ovarian clear cell papillary cystadenoma

MM, malignant mesothelioma.

*Inverted IHC.

^#^The same IHC is omitted.

### 2.4 Treatment and follow-up

After surgical treatment in the local hospital, the patient received six cycles of intravenous chemotherapy with pemetrexed 500 mg/m^2^ combined with cisplatin 70–75 mg/m^2^ in the cancer hospital from May to September 2022. The patient’s postoperative survival period has reached more than 20 months, and no tumor recurrence, metastasis, or ascites was found in regular CT follow-up.

## 3 Discussion and review

### 3.1 Epidemiology and clinical manifestations of OMM

Based on the rarity of this diagnosis, we first conducted a literature review on OMM. MM accounts for 0.3% of global cancer deaths, mainly occurring in the pleura (65%–70%), peritoneum (30%), and pericardium (1%–2%) (10). Peritoneal malignant mesothelioma (PMM) can metastasize to the ovary, but is usually limited to the ovarian cortical surface with microscopic infiltration. MM originating from the ovary is extremely rare. According to the review of relevant literature at home and abroad, only 26 cases of OMM have been reported so far (**Table 2**) (2, 10–22). The 26 OMM patients had an age of onset of 16–74 years, with a median age of 50.5 years. Typical symptoms were abdominal distension and abdominal pain, and some patients had no obvious symptoms and were found by chance during surgery. The tumor diameter was 1–40 cm, and the incidence of bilateral ovarian was 31% (8/26). The pathological types were 14 cases of epithelioid type and two cases of biphasic type. Among the 26 patients, only nine cases had the level of ovarian tumor marker CA125 recorded, of which seven cases were elevated and two cases were normal. Among the seven OMM patients with elevated CA125, three had bilateral mesothelioma lesions. There was no significant correlation between CA125 level and the size of ovarian mesothelioma. The elevated CA125 level may be caused by pelvic inflammation caused by the tumor microenvironment.

**Table 2:** The characteristics of 26 cases of OMM reported internationally.

Case number	Publication (year)	Age (years)	Ovary site	Size (cm)	Clinical manifestations	Histological subtype	Immunohistochemistry	CA125 (U/mL)	Reference number
1	1979	42	Right	1.5	Found during surgery	Epithelioid	NK	NK	(11)
2	1979	74	Left	15	NK	Biphasic	NK	NK	(11)
3	1983	67	Left	9	Found during surgery	Epithelioid	NK	NK	(2)
4	1995	38	Bilateral	NK	Increased abdominal circumference	NK	PCK(+); CEA,B72.3,Leu-M1,Ber-EP4(−)	NK	(12)
5	1995	58	Bilateral	NK	Abdominal pain	NK	PCK(+); CEA,B72.3,Leu-M1,Ber-EP4(−)	NK	(12)
6	1995	71	Left	NK	Abdominal pain	NK	PCK(+); CEA,B72.3,Leu-M1,Ber-EP4(−)	NK	(12)
7	1995	47	Bilateral	NK	Increased abdominal circumference	NK	PCK(+); CEA,B72.3,Leu-M1,Ber-EP4(−)	NK	(12)
8	1995	72	Bilateral	NK	Uterine prolapse	NK	PCK,Ber-EP4(+); CEA,B72.3,Leu-M1(−)	NK	(12)
9	1996	52	Left	3	NK	NK	B72.3,Leu-M1,CEA,Ber-EP4,PLAP(−)	NK	(13)
10	1996	16	Bilateral	10 (left)	Found during surgery	NK	B72.3,Leu-M1,CEA,Ber-EP4,PLAP(−)	NK	(13)
				1 (left)					
11	2000	47	Right	7	Abdominal pain	Biphasic	PCK,Ber-EP4,thrombomodulin,Calretinin,CK5/6(+); CEA,Leu-M1(−)	NK	(14)
12	2000	61	Left	NK	Weight loss	Epithelioid	PCK,Ber-EP4,Calretinin,CK5/6(+); CEA,Leu-M1,thrombomodulin(−)	NK	(14)
13	2000	66	Left	1	Ascites	Epithelioid	PCK,thrombomodulin,Calretinin,CK5/6(+); CEA,Leu-M1,Ber-EP4(−)	NK	(14)
14	2000	66	Right	3	Ascites	Epithelioid	PCK,Ber-EP4,Calretinin,CK5/6(+); CEA,Leu-M1,thrombomodulin(−)	NK	(14)
15	2001	49	Left	NK	fever	Epithelioid	Vimentin,mesothelial(+); Calretinin partly(+); AFP,CD34(−)	82	(15)
16	2006	46	Right	6	Abdominal pain and distension	NK	CK7,CK,mesothelial,Calretinin(+); CK20,Vimentin(−)	>600	(16)
17	2012	55	Bilateral	6 (both sides)	Frequent urination	NK	PCK,CA125,CK7,(+); Calretinin(±); CK20,AFP,CEA,Vimentin(−)	1083	(17)
					Abdominal distension				
18	2015	50	Left	8	Abdominal pain	Epithelioid	CK,vimentin (+); CEA(−)	Normal*	(10)
19	2015	50	Bilateral	11 (left)	Increased abdominal circumference,	Epithelioid	PCK,vimentin,WT-1(+); mesothelial,EMA partly(+);	3023	(18)
				9 (left)	Abdominal distension		D2-40,Calretinin,CK5/6,CK7 focally (+);		
							PLAP,inhibin-α,AFP,CD117,p53,CEA(−);		
							ER positive rate 5%, PR positive rate 10%		
20	2016	39	Right	NK	Abdominal pain	Epithelioid	Calretinin,CK5/6,vimentin,WT-1(+); CEA,P53,ER,PR(-)	Normal*	(19)
21	2017	51	Bilateral	NK	Physical examination found	Epithelioid	WT-1,HBME-1,CK5/6,Calretinin,D2-40,vimentin,CK7,PR(+);	Elevated*	(20)
							CEA,HNF-1β,ER,Ber-EP4(−)		
22	2017	47	Left	NK	Abdominal distension	Epithelioid	WT-1,HBME-1,CK5/6,Calretinin,D2-40,vimentin,CK7(+);	NK	(20)
							CEA,HNF-1β, ER,PR,Ber-EP4(−)		
23	2017	56	Left	3	Abdominal distension	Epithelioid	WT-1,HBME-1,CK5/6,Calretinin,D2-40,vimentin,CK7(+);	NK	(20)
							CEA,HNF-1β,ER,PR,Ber-EP4(−)		
24	2017	69	Right	NK	Abdominal distension	Epithelioid	WT-1,HBME-1,CK5/6,Calretinin,D2-40,vimentin,CK7(+);	NK	(20)
							CEA,HNF-1β,ER,PR,Ber-EP4(−)		
25	2019	35	Left	5	Abdominal distension	Epithelioid	PCK,WT-1,Vimentin,D2-40,CK5/6,Calretinin,IMP-3,HBME-1(+);	1045	(21)
							ER,PR,P53,P16,PAX-8(−)		
26	2021	50	Left	40	NK	NK	CK7,Calretinin,Vimentin,P53,CK8/18,CK19,CK20(+);	233	(22)
							Ber-EP4 weakly(+)		
							CD30,S100,TTF-1,CD56,EMA,CK5/6,WT-1,TFE3(−)		

NK, not known; PCK, pan cytokeratin; PLAP, placental alkaline phosphatase; AFP, alpha fetoprotein; EMA, epithelial membrane antigen; IMP3, insulin-like growth factor II mRNA binding protein 3; TFE3, transcription factor binding to IGHM enhancer 3

*The exact value is not clear.

The combination of IHC selected by previous OMM is different. The most common markers included calretinin, CK5/6, WT-1, D2-40, Vimentin, HBME-1, ER, PR, CEA, Ber-EP4, B72.3, and HNF-1β (**Table 3**), and the first six were mesothelioma markers with high sensitivity. The latter six markers are mostly based on the differential diagnosis of ovarian epithelial tumors, and all of them have high specificity for OMM except Ber-EP4.

**Table 3:** Specific biomarkers of OMM in this patient and 26 previously reported cases.

	Calretinin	WT-1	D2-40	CK5/6	Vimentin	HBME-1	ER	PR	CEA	Ber-EP4	B72.3	HNF-1β	NapsinA	TTF-1	MOC-31	PAX-8	BAP1	GATA3
The patient	+	+	+	+	+	+	−	−	−	+	NK	−	−	−	+	+	Partly+	+
OMM	+	+	+	+	+	+	−	−	−	−	−	−	−	−	−	−	−	+
Sensitivity in 26 case reports	14/15	7/8	5/6	11/12	10/12	4/4	7/7	6/7	19/19	11/17	7/7	4/4	1/1	NK	NK	1/1	NK	NK

The shaded sections represent contradictory results.

Calretinin, CK5/6, WT-1, D2-40, Vimentin, and HBME-1, which had high staining rates and sensitivity of OMM, were all positive in this case. Of the six other markers for differential diagnosis, ER, PR, CEA, and HNF-1β were negative, and B72.3 was not stained, while Ber-EP4 achieved the opposite result. In addition to Ber-EP4, MOC-31, PAX-8, and BAP1 listed in **Table 3** were not consistent with the usual staining results of MM. Can the diagnosis of OMM be established in this patient?

### 3.2 Process of diagnosis establishment based on immunohistochemistry

The local hospital considered that the tumor was a right ovarian mass, and the tumor cells were clear cell type. Thus, the diagnosis was ovarian clear cell carcinoma (OCCC). OCCC showed ER(−), PR(−), WT1(−), and PAX-8(+), all of which were consistent with the immunohistochemistry of this case (23), but HNF-1β and NapsinA, which are more specific for OCCC, were not mentioned (24). However, the diagnosis of OCCC depends on morphological features, such as hyaline degeneration of the papillary axis. OCCC also always had cells with significant nuclear atypia even though most of the cells had only mild nuclear atypia. These were not consistent with the pathological sections in this case. There was also no good explanation for the MM-positive markers Calretinin, D2-40, and GATA-3. Calretinin and D2-40 are two mesothelioma markers recommended by the NCCN Clinical Practice Guidelines in Oncology (25). GATA3 is positive in 58% of mesotheliomas, and its expression in adenocarcinomas of the digestive tract, endometrium, ovary, and prostate is often <10% (26).

Pathological sections from the second hospital, Affiliated Hospital of Tongji Medical College, showed focally well-differentiated papillary mesothelial tumor conformation, with complex papillary structures and stromal invasion of the papillary axis in some areas, which was consistent with ovarian malignant mesothelioma. However, the diagnosis of MM is very difficult. In 2017, the International Mesothelioma Interest Group formulated the diagnostic criteria for MM (3, 27): Immunohistochemical results conform to a set of markers, including two mesothelial positive markers and two other tumor markers considered according to morphology. Re-staining for WT-1, a common marker of MM and OCCC, at the second hospital showed the opposite result, which was positive and supported the diagnosis of MM. The MM markers Calretinin, D2-40, and GATA-3, which were positive in the initial hospital, were also positive. The MM markers CK5/6, HBME-1, and BAP1 were also added. The former two mesenchymal markers were positive in MM, which was consistent with the case. BAP1 is a tumor suppressor gene expressed in normal tissues, showing nuclear denudation in >50% of malignant pleural mesothelioma and two-thirds of PMM cases (28). However, the patient was weakly positive for BAP1. However, BAP1 has high specificity and low sensitivity. Accordingly, the absence of BAP1 in the nucleus strongly supports the diagnosis of MM, but the preservation of BAP1 cannot exclude the diagnosis of MM.

The above MM markers are more than two, and the other tumor markers for morphological considerations are worthy of ovarian clear cell carcinoma related markers. Therefore, OCCC-specific markers were added, such as NapsinA, which was negative, and NapsinA showed cytoplasmic granular staining in clear cell tumors of the ovary (29), which was negative in this case. Unfortunately, Napsin A and HNF-1β are currently two relatively reliable immune antibodies for the diagnosis of OCCC (24). The latter is not shown at this time.

In March of the following year, a pathology consultation was performed in a Shanghai family, and HNF-1β was negative as expected. However, the diagnosis of ovarian clear cell papillary cystadenoma was made based on the positive MOC31 and Ber-EP4. As is known, Ber-EP4 is expressed in epithelial cells but not in mesothelial cells. On the contrary, the statistical analysis of the above 26 cases of OMM reported internationally found that the positive rate of Ber-EP4 was as high as 11/17 (65%), so even if Ber-EP4 is positive, the possibility of OMM cannot be excluded (**Table 3**). MOC-31 is a cell surface protein with carcinogenic characteristics, expressed in healthy epithelial cells and corresponding malignant tumors, often positive in breast cancer, gastrointestinal tumors, and ovarian mucinous carcinoma, not a specific marker of clear cell ovarian tumors, and can also be positively expressed in 5% of PMM (3). Therefore, even if the patient has positive MOC-31 staining, OMM cannot be excluded.

A retrospective review of the patient’s controversial PAX-8 expression was positive, which largely interfered with the judgment of the local hospital. PAX-8 is a specific marker of Mullerian duct epithelium-derived tumors discovered in recent years. During embryonic development, PAX-8 plays a key role in the formation of the thyroid, kidney, part of the central nervous system, inner ear, eye, and Mullerian duct organs. The nuclei of clear cell tumor of the ovary diffusely express PAX-8, so PAX-8 is often used in the differential diagnosis of mesothelioma. The patient also showed strong positive PAX-8 staining; for this reason, some pathological experts did not agree with the diagnosis of mesothelioma. Through a literature search, Chapel and Husain reported 27 cases of peritoneal mesothelioma, of which five cases were positive for PAX-8, including three female patients with diffuse (>50% of the tumor nuclei) staining and two male patients with focal (<50% of the tumor nuclei) staining (30). They believed that PAX-8 could also be positive in MM because PAX-8 and calretinin were co-expressed in the epithelium of the ovary surface, a transitional area known as the tubal–peritoneal junction, where the PAX-8(+) epithelium might sometimes differentiate along the mesothelial lineage, resulting in a series of PAX-8(+) mesothelial lesions. Unfortunately, PAX-8 staining analysis was performed in 1 of the previous 26 cases of OMM (**Table 3**), which also suggested that we could improve PAX-8 staining rate in patients with mesothelioma as much as possible in the future, providing more accurate information for the formulation of future guidelines or consensus.

The onset of mesothelioma was insidious, the clinical manifestations were nonspecific, and the imaging was atypical. It is difficult to distinguish it from other tissues due to its diverse morphology. Although markers with slightly higher specificity and sensitivity in the diagnosis of MM are emerging, it is difficult for any single molecular marker to definitively diagnose mesothelioma. None of the immunohistochemical markers had 100% sensitivity or specificity. The main reason for the inaccurate diagnosis of MM appears to be the use of incomplete immunostaining and incorrect interpretation of staining. A retrospective study on the pathological diagnosis of 92 MM cases in eastern China found that only 56.5% or 52/92 have confirmed diagnosis of cases, 17.4% excluded, and remained uncertain in 26% of cases (31). The results of this study are similar to those of Goldberg in 2006, confirming the diagnosis in 67% of cases and ruling out indeterminate in 13% and 20% of cases (32). Therefore, the problem of MM diagnostic accuracy is not limited to China. For controversial cases, it is necessary to rely on more experienced pathologists to make a second diagnosis, which requires the centralized diagnosis and management of mesothelioma and the formation of a multidisciplinary team to reduce misdiagnosis and missed diagnosis.

Another confusing diagnosis was PMM. Diffuse PMM often involves the ovary, while malignant mesothelioma originating from the ovary is extremely rare. PMM and OMM are easily confused, and the differential diagnosis between them requires observation of the extent of tumor involvement and ovarian condition during operation. In this case, the presence of omental tumor lesions may indicate the seeding of primary ovarian tumors, or they may originate from the peritoneum, which we suggest to be the first possibility. First, the careful examination of the tumor involvement range during the operation could generally only confirm the involvement of the right ovary, and no obvious lesions in the peritoneum were found. Postoperative pathology showed that the tumor was 2 cm in diameter and located in the ovary, and there were nodules with a diameter of 0.5 cm in the greater omentum. Ovarian enlargement is rare in PMM. Even if the tumor metastasized to the ovary, the involvement of the tumor was mostly confined to the serosa and cell cortex, with no invasion of the ovarian parenchyma or only minimal surface invasion and usually a large extra-ovarian space occupying (13).

### 3.3 Clear cell subtype of MM with VHL mutations

The clear cell subtype is a rare variant of epithelioid mesothelioma (25). The most common cause of cytoplasmic hyalinization is the accumulation of large amounts of intracellular glycogen. Another but less common factor is the accumulation of large amounts of lipids, which occurs alone or in combination with glycogen (33). Tumors that are easily confused with clear cell mesothelioma are metastatic renal cell carcinoma, lung adenocarcinoma with clear cell features, and OCCC (6). Inactivation of VHL is common in clear cell renal cell carcinoma (ccRCC), and it has been reported that up to 90% of ccRCC have loss-of-function mutations in VHL (34). Interestingly, this patient presents with the clear cell subtype. The gene testing of this patient showed two missense mutations VHL p.N78S, c.233A>G, VHL p.S65L, and c.194C>T in the first exon of the VHL gene, and both are mutations with potential clinical significance. The product of the VHL gene, pVHL, is involved in the formation of the VCB-CR complex. This complex catalyzes the polyubiquitination of specific proteins and makes them degraded by proteasome. The dysfunction of pVHL leads to the accumulation of downstream hypoxia-inducible factor 1α (HIF 1α) subunits, activates a variety of hypoxia-inducible genes, and promotes the occurrence of tumors under normoxic conditions (35, 36). Screening of the Catalogue of Somatic Mutations in Cancer (COSMIC) database showed that the same missense mutation in this case is highly correlated with renal cancer. For example, the second missense mutation p. S65l has been reported in COSMIC database in 26 cases, all of which were related to the clinical phenotype of renal clear cell carcinoma. Through literature retrieval, a total of seven cases of mesothelioma with VHL gene mutations were reported, including six cases of peritoneal mesothelioma and one case of pleural mesothelioma (37–40) (**Table 4**), and no ovarian mesothelioma has been reported. The types of mutations include frameshift mutations and repetitive mutations. It is interesting to note that even though there are different types of mutations, these patients are characterized by clear cell variant mesothelioma. Therefore, further studies on genetic and epigenetic changes are needed to determine whether there is a relationship between VHL genotype and clear cell phenotype.

**Table 4:** Mesothelioma with VHL mutations.

Publication (year)	Case number	Site	Mutations in genes	Reference number
2019	1	Peritoneal clear cell mesothelioma	VHL exon2, p.F136Nfs 25	(37)
2020	2	Peritoneal clear cell mesothelioma	VHL Y98fs*24	(38)
2021	3	Peritoneal clear cell mesothelioma	VHL exon 1 c.254dupT	(39)
2023	4	Peritoneal clear cell mesothelioma	p.E94Sfs*63	(40)
2023	5	Peritoneal clear cell mesothelioma	p.F136fs*25	(40)
2023	6	Peritoneal clear cell mesothelioma	HD	(40)
2023	7	Pleural clear cell mesothelioma	p.V181fs*18	(40)

## 4 Conclusion

We report the first case of primary ovarian mesothelioma (clear cell variant) with VHL gene mutation in the world. Mesothelioma is common in the peritoneum and the pleural, and primary mesothelioma in the ovary is very rare. In addition, this patient presents with a unique subtype of clear cell variant, which has not been reported so far. Even more interesting is that the VHL gene mutation was also detected in this patient. The disease is easily confused with OCCC, so it is necessary to expand the immunohistochemical marker panel. In a word, both primary ovarian mesothelioma and clear cell variant of mesothelioma with VHL mutations are extremely rare, and a careful diagnosis needs to be made by combining histomorphology, immunohistochemistry, and genetic testing.
